# Modelling and Analysis of the Feeding Regimen Induced Entrainment of Hepatocyte Circadian Oscillators Using Petri Nets

**DOI:** 10.1371/journal.pone.0117519

**Published:** 2015-03-19

**Authors:** Samar Hayat Khan Tareen, Jamil Ahmad

**Affiliations:** Department of Computational Sciences, Research Center for Modeling and Simulation (RCMS), National University of Sciences and Technology (NUST), Islamabad, Pakistan; University of Houston, UNITED STATES

## Abstract

Circadian rhythms are certain periodic behaviours exhibited by living organism at different levels, including cellular and system-wide scales. Recent studies have found that the circadian rhythms of several peripheral organs in mammals, such as the liver, are able to entrain their clocks to received signals independent of other system level clocks, in particular when responding to signals generated during feeding. These studies have found SIRT1, PARP1, and HSF1 proteins to be the major influencers of the core CLOCKBMAL1:PER-CRY circadian clock. These entities, along with abstracted feeding induced signals were modelled collectively in this study using Petri Nets. The properties of the model show that the circadian system itself is strongly robust, and is able to continually evolve. The modelled feeding regimens suggest that the usual 3 meals/day and 2 meals/day feeding regimens are beneficial with any more or less meals/day negatively affecting the system.

## Introduction

### Organisation of the Circadian Clocks

Circadian rhythms are periodic behaviours exhibited by living organisms on a daily basis [1]. These rhythms manifest themselves in apparent behaviour, such as sleeping and feelings of hunger, as well as other physiological functioning and behaviours etc., [2]. They were first observed in the early 18th century by the French astronomer Jean-Jacque d’Ortous De Mairan when he demonstrated the rhythmic opening and closing of the leaves of *Mimosa pudica*, even when the plant was enclosed in a box [3]. Then, in the 20th century, Pittendrigh and Aschoff properly defined the circadian rhythms as a sustained period length under constant conditions, meaning that they are both temperature compensated and independent of environmental effects, but are able to entrain themselves based on certain cues from the environment [3]. In mammals, the circadian system is organised into two major sections, the central circadian clock managed by the suprachiasmatic nucleus (SCN) in the brain, and the peripheral circadian clocks running in different tissues and organs [1]. The primary mode of entrainment for the central clock is via the light signal transmitted through the optic nerve, whereas the peripheral clocks are entrained by a combination of signals originating from both the central clock and localised cues such as xenobiotic stresses and heat shock [1, 4–6].

### Molecular Circadian Oscillators and their Interactions

**Core Circadian Clock.** On the molecular level, the core circadian clock in most cells functions as a negative feedback loop between two protein complexes, CLOCK-BMAL1 (or its homologue NPAS2-BMAL1) heterodimeric transcription factors, and PER-CRY complexes [7–9]. The positive limb of the loop comprises of the transcription of the Period (PER) and Cryptochrome (CRY) proteins by the CLOCK-BMAL1 complex, after which the negative limb of the loop comes into play in the form of the inhibition of the CLOCK-BMAL1 induced transcription by the translated PER-CRY complex, the turnover of which is under strict regulation through E3 ubiquitin ligases [7]. Another linked negative feedback loop is based on the transcriptional activation of the nuclear receptors REV-ERBs and RORs by the CLOCK-BMAL1 complex, where REV-ERBs inhibit the transcription of BMAL1, while RORs activate it [7, 10]. A single cycle of these linked processes takes about 24 hours, enabling the core clock to affect other processes on a proteomic or metabolic level at different times of the day [7].

**Metabolic Circadian Oscillators.** Recent studies have also found that certain metabolites, particularly NAD^+^ shows daily oscillations correlated to circadian rhythms [11]. NAD^+^ is a metabolite, the ratio of which against its reduced form NADH indicates the energy level of the cell. Most of the NAD^+^ in humans is generated by the conversion of nicotinamide via the enzymes Nicotinamide phosphoribosyltransferase (NAMPT) and Nicotinamide mononucleotide adenylyltransferase (NMNAT), a process known as the salvage pathway [12]. NAMPT was found to be the rate limiting enzyme in this pathway [12, 13], which directly correlates with the circadian oscillations of NAD^+^ as NAMPT is also known to be transcriptionally activated by the CLOCK-BMAL1 transcription factor [3]. This allows the circadian clock to regulate other processes such as the activity of the NAD^+^ dependent deacetylase Sirtuin-1 (SIRT1) [3, 14, 15] which shows increased activity in a high cellular NAD^+^: NADH concentration ratio [16].

SIRT1 deacetylase has been shown to be an important component of the cellular machinery, having some prominent proteins such as P53, BMAL1, PER2, PARP1, and HSF1 as its targets [15, 17–21]. Of particular interest with regard to circadian rhythms are its interactions with BMAL1, PER2, PARP1, and HSF1, where SIRT1 is the inhibitor of all except for HSF1, of which it is the activator, through its ability to affect their respective activities via deacetylation. SIRT1 utilises NAD^+^ as a cofactor and, as a stress sensor, is able to confer both pro-survival [19, 22] and pro-apoptotic signals [17] based on stress induced deacetylation of available targets. SIRT1 is mostly produced during the fasting phases in between meals under the influence of CREB, while it is suppressed under the feeding induced production of ChREBP [23]. A number of studies have been carried out which relate restricted feeding (RF) and caloric restriction (CR) induced elevated SIRT1 concentrations with improved health, reduced signs of ageing, and reduced obesity in white mice models [1, 24–26].

**Fig 1:**
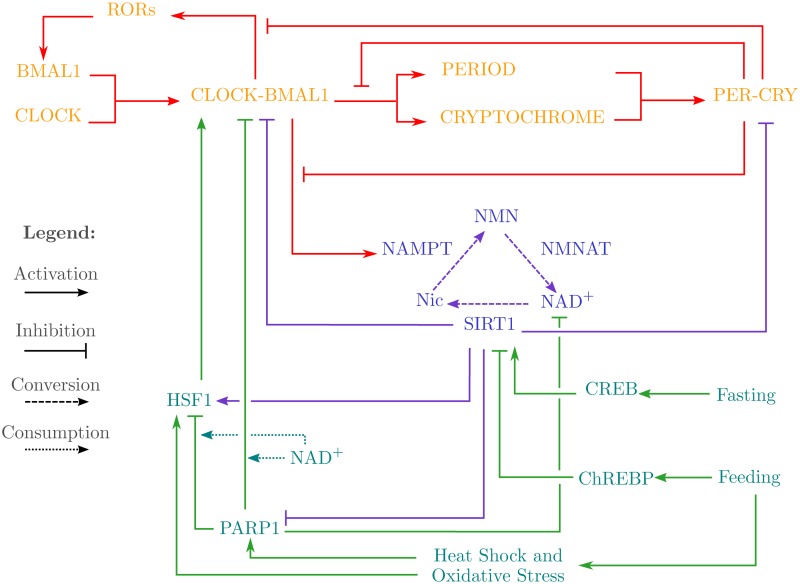
Schematic diagram of the entities and pathways linking feeding-fasting signals to Circadian Clock and Oscillators. The core circadian clock comprises of the entities CLOCK, BMAL1, CLOCK-BMAL1, RORs, PERIOD, CRYPTOCHROME, and PER-CRY. The metabolic arm of the circadian system comprises of the enzymes NAMPT, NMNAT, and SIRT1 with the metabolites Nic, NMN, and NAD^+^. Finally, the entities PARP1, HSF1, CREB, and ChREBP link the feeding and fasting signals to the rest of the circadian system via their interactions.

**Feeding Signals link to Circadain Oscillators.** The studies pertaining to the effects of feeding regimens on white mice have also found that, when localised to peripheral clocks, feeding induces phase shifts in them, such as the phase inversion of the hepatocyte circadian clock [27, 28]. Based on this, it has been suggested that feeding acts as a stronger entrainment cue for the liver cells than the signals from the SCN [29]. These studies also found that during feeding, temperature and oxidative stress signals are transmitted to the peripheral clock via two proteins: Heat Shock Factor 1 (HSF1) and Poly(ADP-ribose) Polymerase 1 (PARP1). Not only has the transcriptional activity of HSF1 been shown to oscillate in a circadian fashion [7], the activity of CLOCK-BMAL1 has also been found to be enhanced in interaction with HSF1 [5], indicating a possible role of temperature and stress induced entrainment of the circadian clock via HSF1 [30]. Likewise, PARP1 has been found to participate in feeding entrainment of the circadian clock by ADP-ribosylating CLOCK, thereby impairing the DNA binding ability of the CLOCK-BMAL1 transcription factor [6, 7]. Of interest is the fact that cellular NAD^+^ levels have been known to plummet during PARP1 activity, since it utilises NAD^+^ as a substrate, negatively affecting the activities of other NAD^+^ dependent proteins and enzymes, such as SIRT1 [31]. As mentioned earlier, HSF1 and PARP1 are also targets of SIRT1, being respectively activated and inhibited, completing a feedback mechanism between the circadian clock and the stress signals induced by feeding. All these pathways and interactions are graphically represented in Fig. 1.

### Computational Modelling of Circadian Rhythms

Although the mentioned studies have established the foundational understanding of the link between the feeding regimens with the peripheral circadian clocks, the complexities and limitations of wet-lab methodologies have localised the inferences to a limited number of proteins and interactions in each study, and not the system as a whole. Here, the application of computational systems biology along with modelling and simulation have allowed the study of abstracted forms of the complete system while preserving the wet-lab established behaviour of the components, in order to find additional behaviours and properties which are not apparent in individual wet-lab studies [32].

**Existing Models.** Over the past decade, a number of studies have modelled the circadian systems utilising different approaches such as differential equations, automata, and Petri Nets. The earliest model of the circadian system was built by Strogatz [33] in the late 1980s. This particular model was based on a simple feedback loop of an activator limb and an inhibitor limb, and did not target specific proteins. Likewise, a relatively recent study [34] also modelled the core circadian system by coupling ultradian oscillators to drive a separate circadian oscillator. Subsequent differential models, such as those of Weimann et al., [35], and Leloup and Goldbeter [36], specifically targeted the proteins comprising the core circadian clock mechanism. Utilising delayed differential equation, Sriram et al., [37] also successfully demonstrated the oscillations of the core circadian clock, which was followed up a few years later by a linear hybrid automata based approach by Ahmad and Roux [38]. A few studies by Matsuno and Miyano ([39] and [40], Chapter 13), utilised the hybrid functional Petri Net framework for the modelling of the core circadian clock, successfully predicting the positive role of RORs in the activation of BMAL1 protein.

**Our Contribution.** Each of the previous models have significantly contributed in the understanding of the circadian system, its function, and its dependence on different components. However, it has also been established in recent studies (as discussed when explaining the metabolic clocks and feeding signals) that feeding regimens have a dominant role in entraining peripheral oscillators, particularly that of the mammalian liver. Our study stands apart from the previously conducted studies in that we not only model the core circadian clock, we also model the recently discovered molecular pathways which link the feeding induced signals to the core circadian clock, successfully modelling and analysing the entrainment of the clock to a periodic environmental cue (feeding). Furthermore, we simulate the effects of different feeding regimens to show how the circadian system is able to entrain to the feeding regimens, and what behavioural changes are observed for each protein involved in the circadian system, proving that the modelled system is capable of transmitting the feeding signals to the peripheral clock for entrainment.

### Structure of the Paper

In this paper we start in the Methods section by explaining the formalisms and frameworks along with their application in systems biology. In the Results section, we present our findings in a step-by-step manner, which are linked and discussed with regards to their biological significance in the Discussion section. We conclude the paper by summarising our findings in the Conclusion section.

## Methods

**Fig 2:**
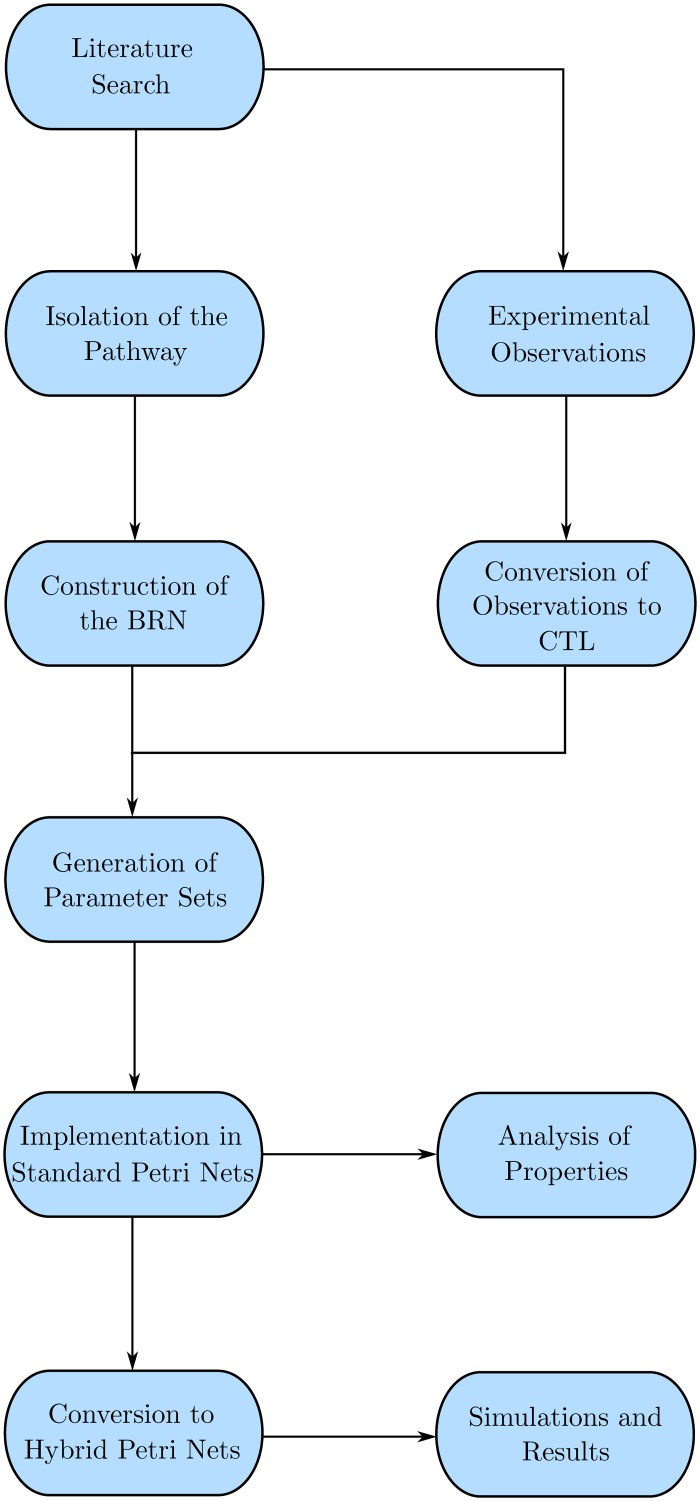
Work Flow Diagram presenting the structure and organisation of the study. The procedure follows after the literature search when a BRN is constructed from the pathway details, upon which the wet-lab experimental observations are applied via CTL Model Checking to generate the set of parameters. The parameters are then modelled as a discrete (Standard) Petri Net for property analysis, after which the system is converted to a Hybrid model for simulations.

Biological systems can be modelled using a number of frameworks such as Differential Equations (ODEs, PDEs, DDEs, PLDEs), Graph theory based networks (Bayesian, Boolean, Logical), Stochastic equations, and Rule based systems to name a few [32]. However, biological systems on the cellular level have complex non-linear dynamics ([41], page 57) which are not properly modelled by mathematical approaches due to the lack of data pertaining to different parameters of the entities involved in the model [32]. Therefore, most of the current graph based modelling frameworks use linear approaches to closely approximate the behavioural dynamics exhibited by the biological systems ([41], page 57). The methodology followed is presented in Fig. 2, and explained below.

### René Thomas’ Kinetic Logic Formalism

In 1970, René Thomas presented a logical formalism [42] that closely approximates the linear differential equations based mathematical models, while keeping the system less complex [43]. Compared to other graph based formalisms, such as the boolean formalism [44, 45], the major advantage of the kinetic logic formalism is the availability of discrete activity threshold levels above ‘1’, as well as asynchronous dynamics [43]. In particular, the asynchronous dynamics allow for the presence of cyclic trajectories representing homoeostatic behaviour, which are absent in the synchronous boolean formalism [44, 45].

**Fig 3:**
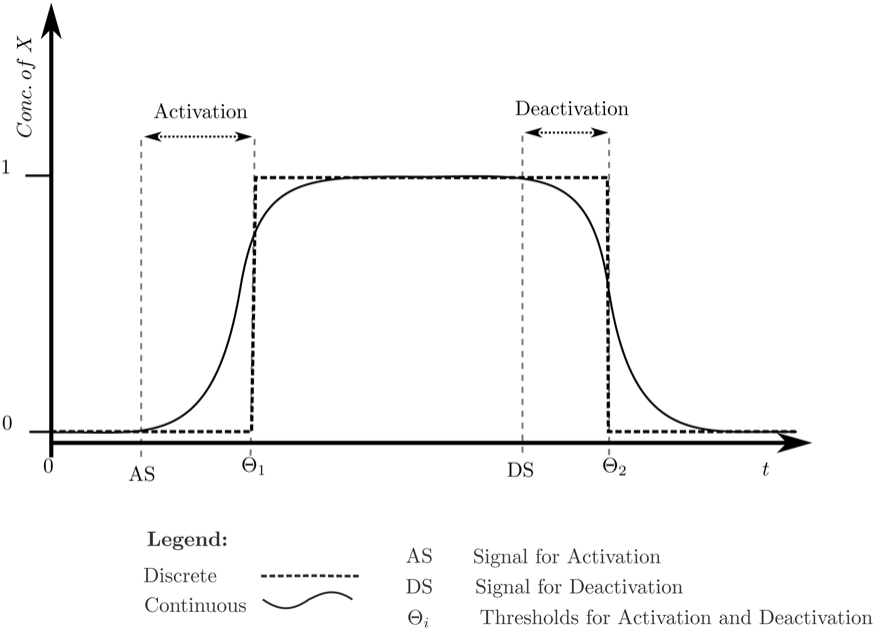
Graph showing the natural continuous, and modelled discrete changes of concentration of an entity X. After the activation or deactivation signal, the protein concentration starts changing immediately while the discrete level remains the same, until a respective threshold is reached.

The kinetic logic formalism qualitatively represents the components of biological regulatory networks (BRNs) as entities, their discrete levels showing their approximate concentrations and threshold dependent interactions. Interactions require that the root entity (the entity which is affecting the other entity) have a minimum level equal to or greater than the threshold requirement of the interaction in order to have an activation (positive), or inhibitory (negative) effect on the level of the leaf entity (the entity which is being affected) [43]. Once the threshold is achieved, the interaction takes place and the level of the leaf entity either increases or decreases, based on the type of interaction, via a step function. The step function keeps the system dynamics qualitative while loosely approximating the sigmoidal behaviour of the biological entities, as shown in Fig. 3. The components and semantics of the formalism, adapted from different studies [43, 46–49], have been defined and explained below. The formal, i.e., mathematical, variants of Definition 3, 4, 5 have been provided in “Formal Definitions” file.

**Semantics of Kinetic Logic Formalism.** The semantics of the René Thomas’ Kinetic Logic Formalism rely on the Graph Theory, particularly the application and computation of directed graphs, along with conditional transitioning between the different states of the graph. The semantics are defined as,

**Definition 1 (Directed Graph)** *A directed graph G(V, E) is a tuple, where:*

- *V is the set of all vertices, and*
- *E is the set of ordered pairs of edges, described as E* ⊆ *V* × *V*

The set of predecessor (resp. successor) entities of an entity *v*_*i*_ ∈ *V* is denoted as *G* ^−^(*v*_*i*_) (resp. *G* ^+^(*v*_*i*_)). The number of predecessors represent the in-degree of the entity, whereas the number of successors represent the out-degree of the entity.

**Definition 2 (Biological Regulatory Network (BRN))** *A biological regulatory network is a directed graph G(V, E) where V is the set of biological entities, and E ⊆ V × V is the ordered set of directed interactions between those entities, and*

- (*j*_*v*_*i*_*v*_*j*__, *η*_*v*_*i*_*v*_*j*__) *is the labelling of the directed edge from the entity v_i_ to the entity v_j_. j_v_i_ v_j__ is the threshold requirement of the edge, while η_v_i_ v_j__* ∈ {+, −} *is the symbol defining the type of interaction:* ‘+’ *being activation, and* ‘−’ *being inhibition*,
- *j_v_i_ v_j__* ∈ {1, 2, …, *r_v_i__*} *where r_v_i__ is less than or equal to the out-degree of v_i_, and*
- *Z_v_i__* = {0, 1, …, *r_v_i__*} *is the set of discrete expression levels of the entity v_i_*.

**Definition 3 (Qualitative States)** *Qualitative states are the configurations of the BRN based on the discrete expression levels of the entities of the BRN*.

Each qualitative state describes one particular configuration of the system, and differs from any configuration in the expression level of at least one entity. All possible configurations of the system constitute the state space of the system, which when represented as a directed graph is known as the state graph of the system/BRN.

The dynamics exhibited by the BRN are dependent on the resources and logical parameters governing the entities of the said BRN. Thus two equivalent BRNs, but with different logical parameters of the entities, can exhibit different dynamics. Resources and parameters are defined as,

**Definition 4 (Resources)** *The availability of an activator, or the absence of an inhibitor of a particular entity is the resource of that entity, with combinations of activators and inhibitors of the entity generating different sets of resources*.

**Definition 5 (Logical Parameters)** *Logical parameters are the K parameters governing the discrete evolution of the BRN*.

The discrete evolution of the entity at a particular discrete level is inferred by the parameter using the available resource set. The entity will approach and attain the value of the parameter, regardless if its current level is greater, or less than the parameter value.

### Isolation of Parameters via Model Checking

As explained earlier, the logical parameters of a BRN govern the dynamics exhibited by the BRN. The logical parameters themselves, however, are dependent on the wet-lab observations and published literature which are not necessarily always available, especially for systems that have a higher number of entities and interactions involved. In recent studies, a new methodology has emerged which allows the generation of desired logical parameter sets via the application of formal methods, in particular model checking [50, 51].

**Model Checking.** Model checking is an exhaustive state space exploration technique that explores all possible states of a given model, testing each state for a required set of properties [52]. This allows complete verification of the system, subject to the level of detail modelled and the set of properties tested. Most model checking techniques employ a subset of Temporal Logic, either Linear-time Temporal Logic (LTL), or Computation Tree Logic (CTL) [53]. In the approach utilised in the work flow of this study, CTL logic has been used due to its inherent catering of a branching state space.

CTL formulae are First-Order predicate logic formulae, which have the general structure Φ = {∀/∃}{◻/◇/*X*}{Φ/*ψ*} consisting of three main parts: a path quantifier, followed by a state quantifier, and lastly the property represented in propositional logic. The structure also allows nesting of path-state quantifier pairs to cater the verification of complex behaviours. The sections are described as:

- The path quantifiers specify the path or trajectory that the property should follow from a current state. Paths or trajectories are series of successive states. The first path quantifier ‘∀’ specifies all paths originating from a current state, while the second path quantifier ‘∃’ specifies at least one path originating from the current state.
- The state quantifiers specify what state the property should hold in. The first state quantifier ‘▫’ specifies global states, that is all states (inclusive of the current state) along the trajectory should hold the property. Likewise, the second quantifier ‘◇’ specifies at least one future state (which can be the current state) should hold the property. Lastly, the ‘*X*’ quantifier specifies the immediate successor state(s) of the current state.
- The last section may, or may not, repeat the path and state quantifiers along with the property. Propositions ‘*p*’, utilising the functions ¬, ∧, ∨, ⇒, ⇔ among others, are used to specify the properties, evaluating to either ‘True’ or ‘False’ on the current state during state space traversal.

**SMBioNet.** SMBioNet is an integrated software which utilises the model checking approach to generate parameter sets against the provided BRN [51]. The source file contains the interactions defining the BRN, optional known parameters, and wet-lab observations encoded in CTL formulae, from which state spaces are generated of all possible parameter sets, which are then verified by the model checker NuSMV via the encoded CTL formulae [50]. Those parameter sets, the state space of which satisfy the CTL formulae, are selected and can then be further screened out through the analysis of their individual parameters or state space to check whether the BRN is exhibiting non-plausible behaviours.

### Implementation of the BRN in Petri Nets

Although software such as GINsim [54] and GenoTech [46] utilise the kinetic logic formalism through discrete automata, the system dynamics remain discrete due to the formalism itself. On the other hand, the Petri Net frameworks can model discrete, continuous and hybrid systems [55], allowing us to model the discrete kinetic logic formalism and then translate it into a hybrid system.

Petri Nets were developed by Carl Adam Petri in 1962 as part of his thesis dissertation, originally to model concurrent processes in technical systems [56]. However, the simplicity of the bipartite framework, along with flexibility in representation, allows Petri Nets to be used in modelling of a number of different systems including chemical reactions, biochemical processes, industrial systems etc., [57]. Petri Nets have already been used to study several regulatory networks and pathways [58–64], for the analyses and predictions pertaining to different system wide behaviours [65]. The primary advantage of Petri Nets lie in their versatility and modularity, allowing for modelling different types of networks (transcriptional, metabolic, protein-interaction and epigenetic etc.) together as a single system for analysis [63].

Our study uses the original discrete Petri net framework to model the kinetic logic formalism based BRN, which is then translated into a Timed Hybrid Petri Net framework to add the continuous dynamics. The frameworks, along with required definitions and properties have been adapted from Chaouiya et al.[65], Blätke et al. [57], and David and Alla [55], and are explained below. The formal variants of Definition 8 and 9 have been provided in “Formal Definitions” file.

**Definition 6 (Directed Bipartite Graph)** *In graph theory, a directed bipartite graph is a special case of directed graph where the set of vertices is itself composed of two distinct subsets such that the subsets have no common elements, and that the edges are always found linking members of the different subsets*.

**Definition 7 (Standard Petri Net)** *A standard Petri Net is a tuple* ⟨*P, T, f, m*_0_⟩ *where*,

- *P is the finite set of places*,
- *T is the finite set of transitions*,
- *f*: ((*P* × *T*) ∪ (*T* × *P*)) → ℕ_≥0_ *is the application of directed arcs, each having a positive integer weight*.
- *m*_0_: *P* → ℕ_≥0_ *is the mapping function which assigns positive integers to the set of places as initial markings*.

**Fig 4:**
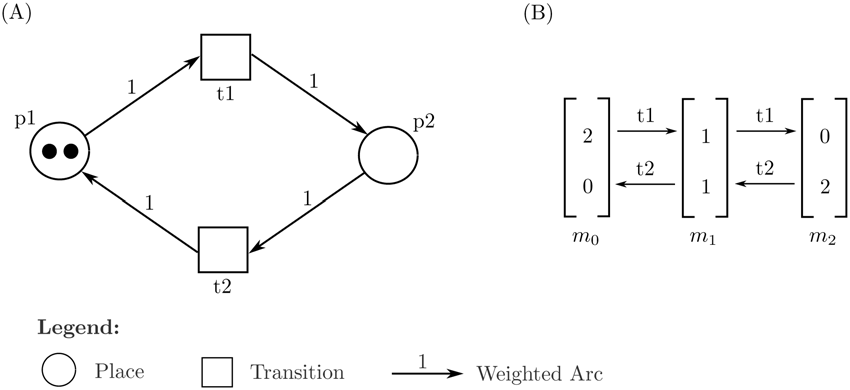
Example of a standard Petri Net. (A) A simple Petri Net model using the standard Petri Net framework. The set *P* = {*p*1, *p*2} is the set of places, *T* = {*t*1, *t*2} is the set of transitions, *f* = {*p*1*t*1, *p*2*t*2, *t*1*p*2, *t*2*p*1} is the set of directed arcs all of which have an arc weight of 1, and *m*_0_ = (2, 0) being the initial marking for the ordered tuple (*p*1, *p*2). (B) The Reachability Graph obtained from the PN from the initial marking *m*_0_. The graph shows three cycles: (2, 0) → (1, 1) → (0, 2) → (1, 1) → (2, 0), (2, 0) → (1, 1) → (2, 0), *and*(1, 1) → (0, 2) → (1, 1); and contains no deadlocks.

Fig. 4(A) illustrates an example along with its system description. For ease in analyses, a further four sets based on the constructed Petri Net are produced:

- °*t* ⊆ *P* is the set of pre-places of a transition *t*,
- *t*° ⊆ *P* is the set of post-places of a transition *t*,
- °*p* ⊆ *T* is the set of pre-transitions of a place *p*, and
- *p*° ⊆ *T* is the set of post-transitions of a place *p*.

In the construction of our Petri Net model, we kept the approach used by Chaouiya et al [61] to model each entity with two places– the primary representing the activated, and the complement representing the deactivated state. The complement state also allows us to introduce capacities to the model to ensure that it follows the same dynamics that SMBioNet inferred. Also, the transitions were used to represent each SMBioNet generated parameter individually so as to ease the analysis of the dynamics of the modelled BRN.

**Semantics and Properties of Petri Nets.** The behaviour and evolution of a Petri Net is defined by the firing of enabled transitions. A transition is said to be enabled, a.k.a live, if the marking of all its pre-places satisfy the respective arc weights between the pre-places and the transition. Once the transition fires, it subtracts the weighted markings from its pre-places and deposits the weighted markings in the post-places. This property is known as firing rule (formally defined in “Formal Definitions” file). Thus starting from an initial marking set *m*_0_, a number of different markings are possible for a given Petri Net, depending on the order of firing, a.k.a firing sequence, of the transitions. These markings are collectively known as the marking graph, or reachability graph with Fig. 4(B) showing the Reachability Graph for the example Petri Net given in (A) of the same figure.

For Petri Nets where some repeated firing of transitions can introduce an infinite number of markings, a special form of the reachability graph, known as Coverability Root Tree, is used where the number of marking is replaced by the symbol *ω* for places containing markings above a certain pre-determined threshold, and are able to cater to infinitely many more.

**Definition 8 (Cycle)** *A cycle is an array of markings formed from the firing of more than one transition, such that the array starts and ends on the same marking*.

**Definition 9 (Deadlock)** *A deadlock is a marking in the Reachability Graph from which no transition is enabled or live*.

**Definition 10 (P-Invariant)** *Also known as conservative component, a P-Invariant is a set of places the weighted sum of markings of which is always constant regardless of the firing of transitions, that is* $\Sigma_{i}^{n}m(p_{i})=constant\;\forall p_{i}\in P,m\in RG$.

**Definition 11 (T-Invariant)** *Also known as repetitive component, a T-Invariant is a firing sequence which returns the marking to the state before firing, that is* $m_{0}\overset{t_{i},\ldots ,t_{n}}{\to }m_{0}$.

In the context of BRNs modelled via Petri Nets, P-Invariants are used to represent different states of an entity, or to group related entities together. Likewise, T-Invariants help in the deduction of cyclic behaviours of the BRN.

**Definition 12 (Liveness)** *Liveness property of a Petri Net dictates that, for any marking of the Reachability Graph, at least one transition is always enabled and able to fire, meaning that the system is always able to evolve*.

**Definition 13 (Reversibility)** *Reversibility property of a Petri Net allows the initial marking m*_0_ *to be reachable from all of the markings of the Reachability Graph*.

The liveness property is falsified with the presence of at least one deadlock in the Reachability Graph. On the contrary, an always live Petri Net of a BRN ascertains the robustness of the system due to its continued evolution. Furthermore, the reversibility property of a modelled BRN ensures that the entities of the BRN are always able to reinitialise the system to a some starting or initial point, from any point in the dynamics of the system.

**Continuous Dynamics.** Standard Petri Nets are useful for property analyses, but are limiting since the dynamics of the system remain discrete. To analyse the continuous dynamics, we utilise an extension of the standard Petri Nets, namely Timed Hybrid Petri Nets, which are formally defined as,

**Definition 14 (Timed Hybrid Petri Net (THPN))** *A Timed Hybrid Petri Net is a tuple* ⟨*P, T, f, h, m*_0_, *tempo*⟩ *where*,

- *P is the finite set of places*,
- *T is the finite set of transitions*,
- *f*: ((*P* × *T*) ∪(*T* × *P*)) → ℝ_≥0_ *is the application of directed arcs with a positive real number weight*,
- *h*: *P*∪*T* → {*Disc, Cont*} *is the hybrid function assigning the type ‘discrete’ (P^D^, T^D^) or ‘continuous’ (P^C^, T^C^) to each vertex*,
- *m*_0_: *P* → ℝ_≥0_ *is the initial marking of positive real values of the places*,
- *tempo*: *T* → {ℚ_≥0_∣*t* ∈ *T* ^*D*^}∪{ℚ∣*t* ∈ *T* ^*C*^} *is the assignment function which assigns discrete delays to discrete transitions and rates to continuous transitions*.

In its simplest form, the rate of the continuous transition can be the inverse of an associated delay [55], and can be used in the mass action kinetics equation of the form *dX*/*dt* = *rate* × ∏*m*(°*t*) for a place *X* ∈ *t*° and transition *t* ∈ *T* ^*C*^ [57]. This allows for continuously evolving dynamics as the rates of the transitions become dependant on the markings of their pre-places i.e., the system responds strongly to stronger concentrations of stimuli and vice versa.

### Software and Modelling Data

As mentioned in the Methods section, SMBioNet [50] was used to calculate the logical parameters. By default, it utilises the NuSMV symbolic model checker [66] for satisfying the CTL formulae. All Petri Net models were constructed using Snoopy 2 [67], and the properties were analysed on the standard Petri Net using Charlie [68]. The SMBioNet output has been provided as “SMBioNet Output”, while the Snoopy models with Charlie analyses have been provided as “Standard PN Model” and “THPN Models” supplementary files.

## Results

In this section we have presented our results in a procedural manner and show how the previous result ties in with the next framework or formalism, starting from the isolation of the BRN till the simulation results of the regimen scenarios.

### Construction of the BRN

The primary concern plaguing computational approaches is the complexity of the system being modelled. This complexity restricts the modelling of all components of the system, especially if the system is large, since the analysis either results in a state space explosion, or complex and tedious to manage results. In a recent study, Richard et al.[69], calculated that the SMBioNet software is only able to work with smaller entity sizes, typically less than seven, citing the exponential complexity of (2^*n*^)^(2^*n*^)^ as the primary concern for this limitation.

To cater to this limitation, we reduced the schematic diagram, given in Fig. 1, to six entities whilst preserving the behaviour of the system as much as possible. This was achieved by the following changes:

- The mediated effects of the Feeding signal on HSF1, PARP1, and SIRT1 were represented directly.
- The effect of Fasting on SIRT1 was abstracted into the logical parameters represented in the Results section.
- The individual BMAL1 and CLOCK1 entities, along with the CLOCK-BMAL1 complex and ROR loop were represented together as a single entity CLOCK-BMAL1.
- Likewise, PERIOD and CRYPTOCHROME proteins, along with their complex PER-CRY were simply represented as the PER-CRY complex. Their interactions with CLOCK-BMAL1 mediated transcriptions were shown as an inhibition of the CLOCK-BMAL1 complex.
- Finally, the salvage pathway of NAD^+^ was completely abstracted, and represented as the direct activation of SIRT1 by the CLOCK-BMAL1 complex, on the basis of the NAMPT dependent oscillations of SIRT1 explained in the Introduction section. In suit, the depletion of NAD^+^ by the activity of PARP1 was abstracted as the inhibition of SIRT1 by PARP1.

**Fig 5:**
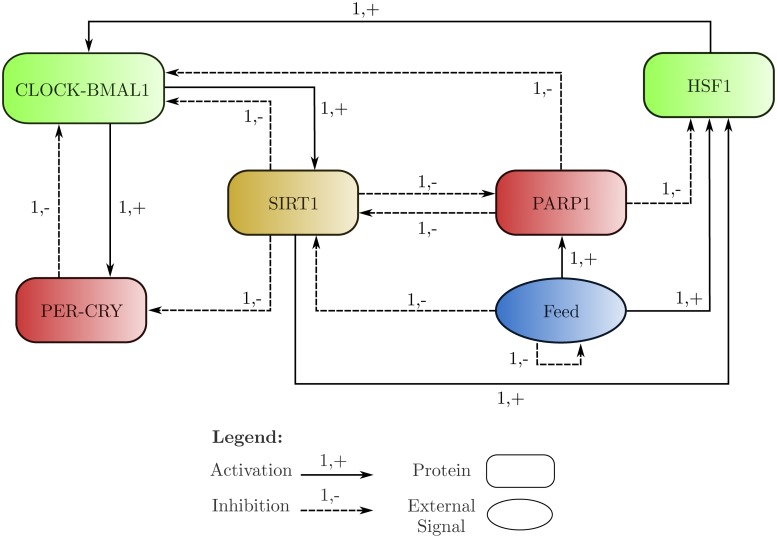
The BRN generated by abstracting the entities and pathways of the schematic diagram shown in Fig. 1. The proteins CLOCK-BMAL1 and HSF1 are showing activatory interactions, while PER-CRY and PARP1 are showing inhibitory interactions only. SIRT1 is showing both activatory and inhibitory interactions. The Feeding signal, being an abstracted environmental cue, is represented as an oval.

The BRN is represented in Fig. 5. The behaviours of these interactions were conserved by assuming a slightly different approach to the understanding of the entity levels– the level of an entity represents the available concentration of the entity performing the represented function, and not the total cellular concentration of that entity. Thus the inhibition of CLOCK-BMAL1 by PER-CRY does not represent the depletion of cellular levels of CLOCK-BMAL1, but rather the depletion of the levels of CLOCK-BMAL1 that are able to perform the function of transcriptional activation of PER-CRY and SIRT1. This approach is applicable to all entities of the BRN, except for the external signal of Feeding.

### Isolation and Selection of Logical Parameters

The BRN presented in Fig. 5 was then used in SMBioNet to generate the parameter models of the BRN. The following CTL formulae were used in conjunction in SMBioNet to screen the generated parameter models:

$$\Phi_{1}=Init\to \forall X(\exists ◇(Init))$$ (1)

$$\Phi_{2}=(CB=1\wedge PC=0)\to \exists X(CB=1\wedge PC=1)$$ (2)

$$\Phi_{3}=(CB=1\wedge PC=1)\to \exists X(CB=0\wedge PC=1)$$ (3)

$$\Phi_{4}=(Feed=1\wedge HSF1=0)\to \exists X(Feed=1\wedge HSF=1)$$ (4)

$$\Phi_{5}=(Feed=1\wedge PARP1=0)\to \exists X(Feed=1\wedge PARP=1)$$ (5)

$$\Phi_{6}=(Feed=1\wedge SIRT1=1)\to \exists X(Feed=1\wedge SIRT1=0)$$ (6)

Here CB is the abbreviation for CLOCK-BMAL1, PC is the abbreviation for PER-CRY complexes, and *Init* is the initial condition where all entities are at level ‘0’. Formula 1 specifies that the initial configuration of the system is cyclic, and is reachable from its successors. Formulae 2 and 3 enforce that the core clock is always able to function. Likewise, formulae 4, 5, and 6 enforce that the Feeding signal will always interact with its targets.

Table 1 shows the parameters that were used to construct the Petri Net model. The allowed values show the parameter values that were allowed to SMBioNet for generation of parameter sets. Some of these allowed parameters values were restricted to a single level based on the criteria that if an entity has no activator and is being inhibited by at least one of its inhibitors, then the parameter for that entity is restricted to ‘0’ as in the case of the parameter *K*_*CB*_{}. Likewise, if an entity has at least one activator as its resource, and is not being inhibited by any inhibitor, then its respective parameter is restricted to ‘1’ as in the case of the parameter *K*_*CB*_{*HSF*1, *PARP*1, *PC, SIRT*1}. A few parameters, which had both the activators and inhibitors of the entity present, were also restricted based on wet-lab evidence or dynamics. One example is the parameter *K*_*SIRT*1_{*Feed, PARP*1} in which neither the inhibitors nor the activators are present. However, this parameter was restricted to ‘1’ citing the CREB mediated activation of SIRT1 induced by fasting, as shown in Fig. 1. Such restrictions allow us to still be able to model the behaviours which are usually lost during the abstraction of the system.

**Table 1:** Table showing the parameters, their respective resource sets, and the values that were allowed in SMBioNet, generated by SMBioNet in the parameter sets, and finally selected for modelling in Petri Nets.

**Parameters**	**Resources**	**Values**		
		**Allowed**	**Generated**	**Selected**
*K*_*CB*_	{}	0	0	0
	{HSF1}	0, 1	0	0
	{PARP1}	0	0	0
	{PC}	0	0	0
	{SIRT1}	0	0	0
	{HSF1, PARP1}	0, 1	0	0
	{PARP1, PC}	0	0	0
	{PARP1, SIRT1}	0	0	0
	{HSF1, PC}	0, 1	0, 1	0
	{PC, SIRT1}	0	0	0
	{HSF1, SIRT1}	0, 1	0	0
	{HSF1, PARP1, SIRT1}	0, 1	0	0
	{HSF1, PARP1, PC}	0, 1	0, 1	1
	{HSF1, PC, SIRT1}	0, 1	0, 1	0
	{PARP1, PC, SIRT1}	1	1	1
	{HSF1, PARP1, PC, SIRT1}	1	1	1
*K*_*PC*_	{}	0	0	0
	{CB}	0, 1	1	1
	{SIRT1}	0	0	0
	{CB, SIRT1}	1	1	1
*K*_*SIRT*1_	{}	0	0	0
	{CB}	0	0	0
	{Feed}	0	0	0
	{PARP1}	0	0	0
	{CB, Feed}	0, 1	0, 1	0
	{Feed, PARP1}	1	1	1
	{CB, PARP1}	0	0	0
	{CB, Feed, PARP1}	1	1	1
*K*_*PARP*1_	{}	0	0	0
	{Feed}	1	1	1
	{SIRT1}	0, 1	0, 1	0
	{Feed, SIRT1}	1	1	1
*K*_*HSF*1_	{}	0	0	0
	{Feed}	0, 1	1	1
	{PARP1}	0	0	0
	{SIRT1}	0, 1	0, 1	0
	{Feed, PARP1}	0, 1	1	1
	{PARP1, SIRT1}	1	1	1
	{Feed, SIRT1}	0, 1	1	1
	{Feed, PARP1, SIRT1}	1	1	1
*K*_*Feed*_	{}	0	0	0
	{Feed}	1	1	1

Of these allowed parameters, SMBioNet generated 26 parameter sets which were satisfying the CTL constraints given in equations 2–7. These sets were differing on 6 parameters, shown as Generated Values in Table 1. Thus the selection of a single parameter set proceeded by selectively fixing the value of each of these parameters in the following order:

1. *K*_*HSF*1_{*SIRT*1} was fixed to ‘0’ under the assumption that presence of PARP1 will be able to deplete cellular NAD^+^ levels, thus stopping SIRT1 from activating HSF1 [31].
2. *K*_*PARP*1_{*SIRT*1} was fixed to ‘0’ because SIRT1 is constantly active during the fasting periods and will inhibit PARP1, whereas PARP1 relies on the feeding signal for its activation and depletion of cellular NAD^+^ levels [6, 23].
3. *K*_*SIRT*1_{*CB, Feed*} was fixed to ‘0’ because SIRT1 levels are known to be reduced during PARP1 activity [31].
4. *K*_*CB*_{*HSF*1, *PC, SIRT*1} was fixed to ‘0’ as PARP1 is known to reset the core circadian clock [70]. This also restricted the parameter *K*_*CB*_{*HSF*1, *PC*} to ‘0’ as well.
5. *K*_*CB*_{*HSF*1, *PARP*1, *PC*} was fixed to ‘1’ as core circadian clocks show oscillations throughout the day, including the fasting periods.

The selected parameter set was then used in the ensuing construction of the discrete Petri Net model of the BRN.

### Standard Petri Net Model and Property Analysis

As discussed in the Methods section, the BRN of the model was constructed by representing each entity with two places– one modelling the presence of the entity, and the other modelling its absence. This allowed us to represent the absence of inhibitors as resources by originating the respective arcs from the place modelling their absence. This complementary place modelling is also being used to impart capacities to restrict the maximum achievable levels of the entities in accordance with the SMBioNet model.

Each parameter of respective entities was modelled as an individual transition directing an arc towards or away from the places of the entities. Thus the source transitions °*p* (resp. sink transitions *p*°) of the place modelling the presence (resp. absence) of a particular entity *X* represent those parameters which allow *X* to achieve a level > 0. Likewise, the sink transitions *p*° (resp. source transitions °*p*) of the place modelling the presence (resp. absence) of the entity *X* represent those parameters allowing *X* to achieve the level 0.

**Fig 6:**
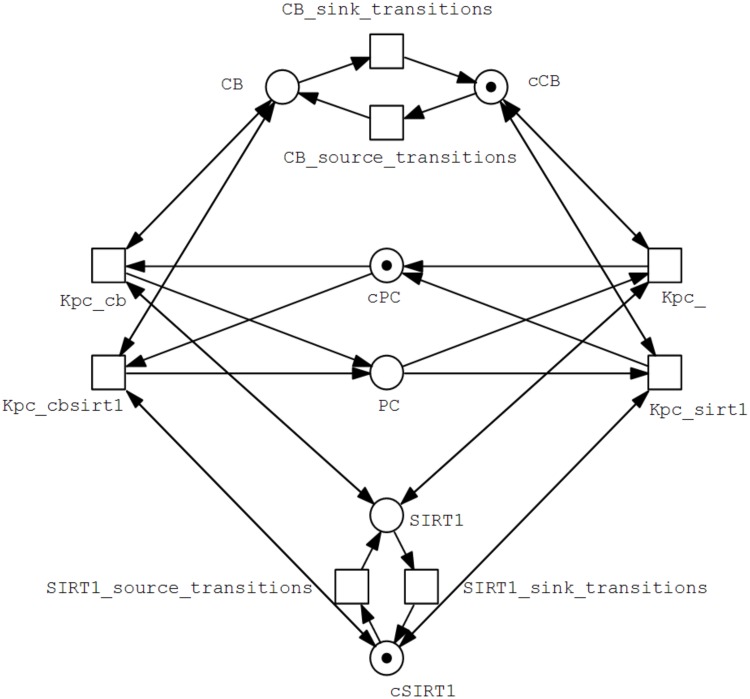
Partial discrete Petri Net model of the BRN. This part represents the complete Place and Transition sets of the entity PC (*P* = {*PC, cPB*}, *T* = {*Kpc*_, *Kpc*_*cb, Kpc*_*sirt*1, *Kpc*_*cbsirt*1}), where the place *PC* models the presence of PC in the system, and *cPC* models its absence. PC has four parameters, *K*_*PC*_{*CB*}, *K*_*PC*_{*CB, SIRT*1} ∈ °*p*, and *K*_*PC*_{}, *K*_*PC*_{*SIRT*1} ∈ *p*° for *p* = *PC*. Highly abstracted sets of the influencers of PC (CB and SIRT1) are also represented simply as the respective presence and absence places, along with generic source and sink transitions. As CB is the activator of PC, the transitions where it is in the resource set of PC link to the place *CB*, and the transitions where it is absent from the resource set link to the place *cCB*. On the other hand, this pattern is inverted in the case of SIRT1 as it is an inhibitor of PC, and thus the transitions where it is in the resource set of PC link to the place *cSIRT*1, while the transitions where it is absent from the resource set link to the place *SIRT*1 instead.

The complete discrete Petri Net model is difficult to illustrate or represent as a figure due to its complexity and large number of arcs. Instead, a partial model comprising of a single entity PC along with highly abstracted forms of its influencers CB and SIRT1 is represented as an example in Fig. 6 to illustrate how the model is constructed.

**Fig 7:**
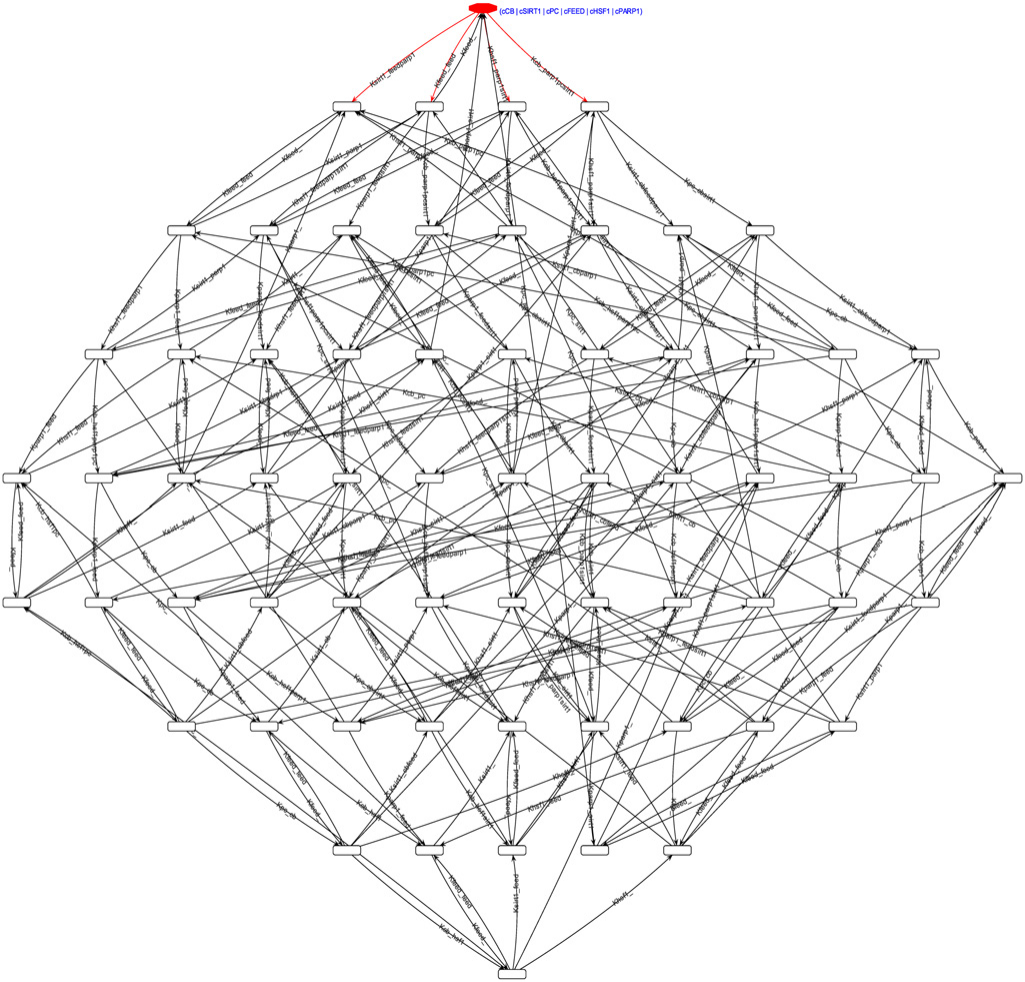
The generated reachability graph of the complete discrete Petri Net model. The order of the tuple in each marking is ⟨CB, SIRT1, PC, Feed, HSF1, PARP1⟩. The graph was generated from the initial marking *m*_0_ = (0, 1, 0, 1, 0, 1, 0, 1, 0, 1, 0, 1), shown at the top. It consists of 64 unique markings and 224 marking transitions, and is itself a single strongly connected component.

**Reachability Graph.** The generated reachability graph of the complete discrete Petri Net model of the system in the order ⟨CB, SIRT1, PC, Feed, HSF1, PARP1⟩, shown in Fig. 7 and initialising from the marking *m*_0_ = (0, 0, 0, 0, 0, 0) (*m*_0_ = (0, 1, 0, 1, 0, 1, 0, 1, 0, 1, 0, 1) if the respective complementary places are included), shows a total of 64 unique markings including *m*_0_, with 224 transitions between the markings. The complete graph is a single strongly connected component, meaning that each pair of markings is reachable from one another.

**Properties of the System.** The strong connectedness of the reachability graph indicates that all marking trajectories of the discrete Petri Net model always end up in a cycle. This implies that the system is free of any deadlocks, and that the complete system is both live and reversible. This implication is also backed up by the T-invariant analyses, wherein every pair (°*p*_*x*_*i*__, *p*°_*x*_*j*_)_ for the place representing the presence of an entity *X* is a T-invariant. The P-invariant analyses couple the presence and absence places of each entity as the invariant, preserving the level capacities of the entities.

### Hybrid Petri Net Model and Simulation Results

The Standard Petri Net model was exported to a Hybrid Petri Net model wherein the discrete places and transitions were converted to continuous places and transitions, respectively. Consequently, the complementary places along with their respective arcs were removed to clear any restrictions imposed by them on the continuous dynamics of the system. Finally deterministic (timed) transitions were used to model the periodic feeding signals.

As mentioned in the Methods section, the rate function used in the continuous transitions utilise the mass action kinetics equations. Given that the entrained circadian oscillations have a period length of about 24 hours [71], the rates of the entities were manually adjusted to produce an approximate 24 hour periodic behaviour of the entities against an assumed normal feeding regimen of 3 meals a day (0800 hrs breakfast, 1400 hrs lunch, 2000 hrs dinner) followed by a 12 hour overnight fast. The rates were associated with the proteins only since the feeding signal is being modelled as a deterministic subsystem, and are listed in Table 2.

**Table 2:** Table showing the rates used for the continuous transitions of each entity. The concentration increase rate specifies the rate for the transitons *t* ∈ °*p*, and the concentration decrease rate specifies for *t* ∈ *p*°, for place *p* representing the entity in the system.

**Entities**	**Rate Values**	
	**Conc. Increase**	**Conc. Decrease**
CLOCK-BMAL1	1/5	1/45
PER-CRY	1/5	1/15
SIRT1	1/45	2/15
PARP1	1/2	1/15
HSF1	1/10	1/15

**Fig 8:**
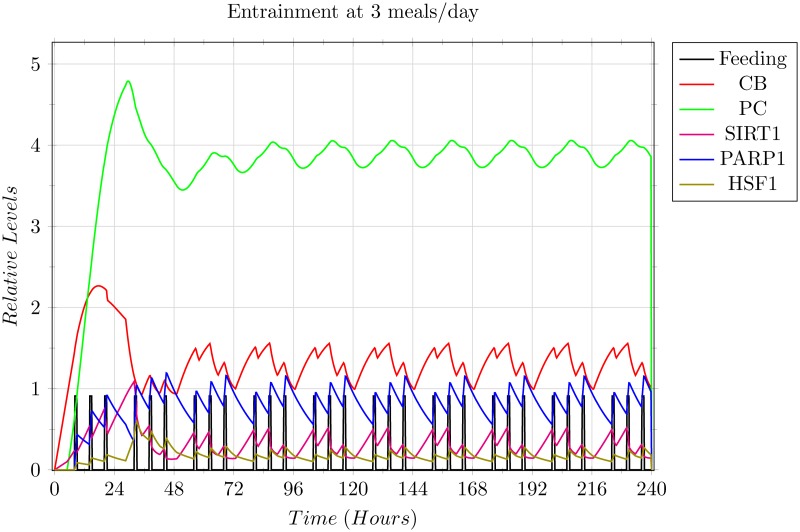
Simulation results of the 3 meals/day entrainment base scenario. Each grid box shows 1 complete day on the x-axis, and a complete level on the y-axis. The graph shows the oscillations of the entities in accordance with the 0800 hrs breakfast, 1400 hrs lunch, and 2000 hrs dinner, with an overnight fast of 12 hours, for a duration of 10 days. The entities were able to entrain on the third day, and continued the periodic behaviour from then on. All entities are utilising their respective rate values given in Table 2, except for the Feeding signal which is modelled as a periodic discrete signal, shown as the vertical black pillars. The number of meals, and their timings was assumed to be the prevalent regimen, and were thus used as the base scenario to model other differing regimen scenarios.

The resulting 3 meals/day entrainment graph (Fig. 8), shows local minima and local maxima during the oscillations of the entities, attributed to the feeding signals received at that time point, but the overall behaviour of the entities is preserved to show the 24 hour periodic oscillations. This pattern was used as the base scenario for entrainment and the simulations showing other feeding regimens utilise this base scenario for the initial entrainment.

**Fig 9:**
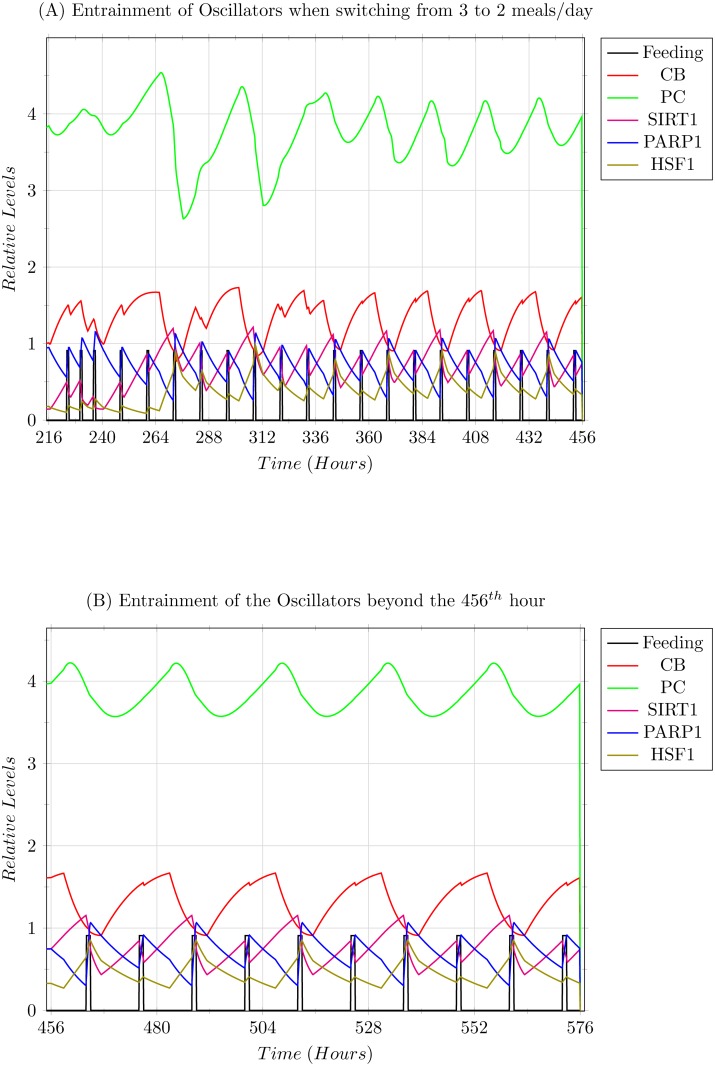
Simulation results of the 2 meals/day entrainment explained in Scenario 1. (A) The graph shows the oscillations of the entities in accordance with the 0800 hrs breakfast, and 2000 hrs dinner, with an overnight fast of 12 hours. (B) The graph shows the oscillation beyond the 456^*th*^ hour, in which the PER-CRY (PC) protein complex converges into its rhythm.

**Scenario 1: Modelling 2 meals/day entrainment.** In this scenario, the feeding regimen was shifted to 2 meals/day starting from the 11^*th*^ day. The oscillators were able to entrain, but showed a 12 hour shift in the entrained behaviour. The initial switch is shown in Fig. 9 (A), where as (B) shows the convergence of the PC complex, which was the slowest to entrain in this scenario.

**Fig 10:**
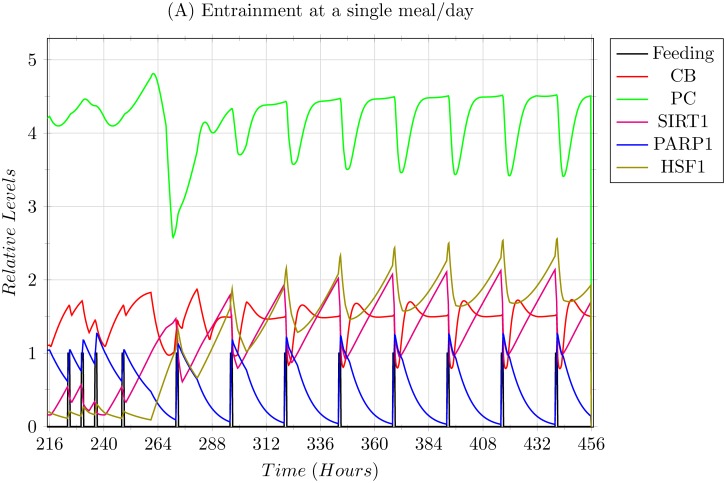
Simulation results of a single 0800 hrs breakfast/day entrainment explained in Scenario 2. The system was able to entrain but showed high levels of stress via higher expressions of SIRT1 and HSF1.

**Scenario 2: Modelling 1 meal/day entrainment.** In this scenario, the 3 meals/day entrainment was subjected to a sudden shift to a single 0800 hrs breakfast/day regimen (Fig. 10). The system was able to adapt and entrain, but shows high levels of stress as apparent with the high levels of both SIRT1 and HSF1. Another prominent aspect is the trajectories of CLOCK-BMAL1 (CB) and PER-CRY (PC) complexes which achieve a temporary equilibrium during their oscillations, and are only disturbed via the feeding signal, giving merit to the observation that feeding indeed resets the circadian clock.

**Fig 11:**
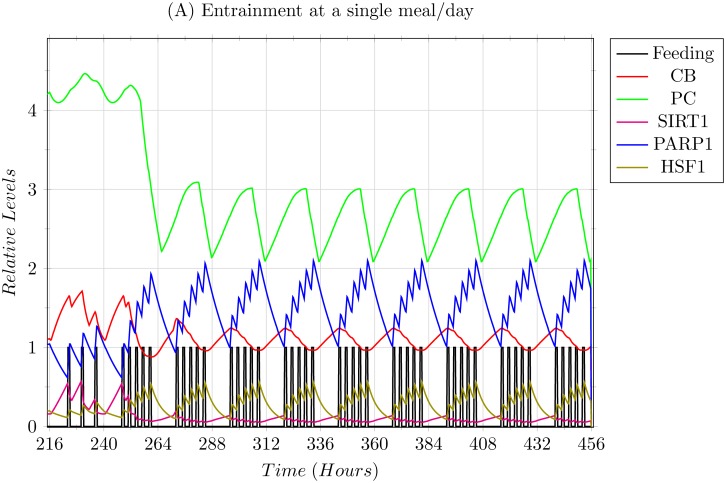
Simulation results depicting the over feeding scenario with 5 meals/day. The meals were simulated on 0800 hrs, 1100 hrs, 1400 hrs, 1700 hrs, and 2000 hrs, along with the 12 hour overnight fast. The results indicate an over expression of PARP1, with suppressed expressions of almost all entities, other than HSF1.

**Scenario 3: Modelling 5 meals/day entrainment.** This scenario is the complete opposite of Scenario 2, and thus models the case of over feeding via the 5 meals/day regimen based on the timings 0800 hrs, 1100 hrs, 1400 hrs, 1700 hrs, and 2000 hrs, with a 12 hour overnight fast. The simulation results, shown in Fig. 11, indicate that over feeding suppresses the expression of the core circadian clock, and just about diminishes any expression of SIRT1.

## Discussion

In this section, we have discussed the inferences derived from the results obtained in the previous section and speculated over the possible long term effects that these results might imply. We start by discussing the results pertaining to the robustness of the circadian system.

### Robustness of the Circadian System

In all the various wet-lab studies that have been conducted on white mice models, the mice were able to entrain to the change in the feeding regimens, specifically in cases of restricted feeding (RF) [1, 28], caloric restriction (CF) [26, 72, 73], and inversion of regimen timings to the day time [6, 7] (mice are nocturnal mammals). Provided that most of the proteins observed showing circadian oscillations have respective homologous proteins in *Homo sapiens*, the robustness is expected in the human systems as well. This was confirmed in our model via the result that, (i) there weren’t any deadlock markings in the reachability graph indicating that the system is always able to evolve, which together with the second reason that (ii) the whole reachability graph is a single strongly connected component, implies that (iii) not only is the system completely live and always able to evolve, but also reversible, meaning that the system always has the option to, for want of a better term, ‘reboot’ itself to an arbitrary starting point from any other point in its oscillations. The results from the T-invariant analysis also back up this inference, leading us to the next inference that this robustness plays a vital role in the entrainment of the circadian rhythms to external signals, such as the feeding signal modelled in this study, allowing the system to adapt to any perceived realistic scenario (barring extreme cases such as life threatening starvation etc.).

### Effects of the Feeding Signal on Circadian Entrainment and Oscillator Expression

As explained in the Results section, the 3 meals/day feeding regimen with timing 0800 hrs breakfast, 1400 hrs lunch, and 2000 hrs dinner was assumed to be the prevalent regimen and was thus used as the baseline for circadian entrainment and comparison with other regimens. It was observed that in the first scenario, where the switch was made to 2 meals/day, the oscillators did not show much variation from their original oscillatory pattern, with the exception of SIRT1 and HSF1 which were expressed slightly more than previously, and that the oscillation of the entities showed a 12 hour forward shift in the pattern. This expression of SIRT1 corresponds with the observations that SIRT1 is expressed more during the fasting periods, and is actually a preferred situation since SIRT1 has already been linked with anti-ageing and longevity roles when limitedly expressed in a higher concentration [1, 12, 17, 24–26, 74]. Likewise, HSF1 expression has also been found to be active in a cytoprotective manner, backing up SIRT1 in this domain [17, 75, 76]. These inferences thus also impart merit to some of the different patterns of fasting observed globally in different cultures and regions [77].

The two extreme scenarios of feeding regimens given in the Results section, Scenario 2 being that of under feeding, and Scenario 3 being that of over feeding, show how varied the effects of feeding regimens are based on the frequency of the feeding signals. In Scenario 2, restricting the regimens to a single meal per day drastically affected the circadian rhythms, showing increased stress in the form of over expression of HSF1 and SIRT1. The over expression of SIRT1 in turn introduced some limiting effects on the core circadian clock, apparent by the partial equilibrium levels achieved by the CB and PC complexes during or near the peak expression of SIRT1 as shown in Fig. 10. This is presumed as dangerous since not only damped oscillations of the core clock will affect targets of the core clock other than the ones modelled in our system, but high stress is known to induce SIRT1 to promote pro-apoptotic signals instead of pro-survival signals [17].

In Scenario 3, the case of over feeding, PARP1 was shown to be highly expressed, affecting and dampening the core clock oscillations drastically. Apart from the suppression of the core clock, it was also observed that the expression levels of SIRT1 were almost negligible, thus devoiding the system of the established beneficial role of SIRT1. This was followed by slightly higher expression of HSF1 due to the repeated oxidative stress, indicating that the over feeding regimen was frequently putting the system under stress on a cellular level through reactive oxygen species, slowly eroding the cellular machinery and processes– thus ageing the system.

Collectively, the entrainment to periodic feeding regimens also provides evidence that the modelled BRN, although just comprising of the hepatocyte clock, is enough to translate the feeding induced signals to entrain itself. This indicates that the modelled BRN represents ample infrastructure to retain localised entrainment independent of the central clock, as found in wet-lab studies [27–29].

## Conclusion

Based on the results and the discussion, a two part conclusion is drawn that, (i) the circadian system and the interactions of the circadian oscillators are highly robust, allowing the system to continue evolving in any provided realistic and non-fatal situation, and (ii) of the modelled feeding regimens, 3 meals/day and 2 meals/day have been shown to be beneficial with the advantages of more expressed SIRT1 in the 2 meals/day regimen giving it the edge in terms of longevity. Any less or any more meals/day in the regimens dramatically affect the circadian oscillators and their expression, with the harmful effects far outweighing the perceived need of such extreme regimens.

## Supporting Information

S1 Materials Formal Definitions. (PDF)

S2 Materials SMBioNet Output. (OUT)

S3 Materials Standard PN Model. (SPEPT)

S4 Materials THPN Models Scenario B. (SPHYBRID)

S5 Materials THPN Models Scenario 1. (SPHYBRID)

S6 Materials THPN Models Scenario 2. (SPHYBRID)

S7 Materials THPN Models Scenario 3. (SPHYBRID)
