# Supplementary material for: Modelling and Analysis of the Feeding Regimen Induced Entrainment of Hepatocyte Circadian Oscillators Using Petri Nets
Source: PLoS One. 2015 Mar 19;10(3):e0117519. doi: 10.1371/journal.pone.0117519 (PMC4366204; doi:10.1371/journal.pone.0117519)
Supplement: S1 Materials — (PDF) [file pone.0117519.s001.pdf]

Formal Definitions

Qualitative States

Qualitative states are the configurations of the BRN based on the discrete expression levels of the entities of the BRN. A set 'S' of all qualitative states of a BRN G(V,E) is defined as, $S = \Pi_{v_i \in V} Z_{v_i}$.

A tuple $s \in S$, defined as $(s_{x_{v_i}})\ \forall v_i \in V$, represents one particular configuration of the system, with x_{v_i} representing the expression level of the entity v_i. Two configurations $s_m \in S$ and $s_n \in S$ for $m \neq n$ differ in at least one x_{v_i}. All possible configurations of the system constitute the state space of the system, which when represented as a directed graph is known as the state graph of the system/BRN.

Resources

In a given configuration of a BRN, the set of resources $Q_{x_{v_j}}$ *for any entity* $v_j \in V$ *are defined as* $Q_{x_{v_j}} = \{v_i \in G^-(v_j) | (x_{v_i} \geq j_{v_i v_j} \wedge \eta_{v_i v_j} = +) \vee (x_{v_i} < j_{v_i v_j} \wedge \eta_{v_i v_j} = -)\}$. *This implies that an inhibitor* $v_i \in G^-(v_j)$ *is treated as resources of* v_j *iff it is absent in the given configuration.*

Logical Parameters

The set of logical parameters governing the dynamics of a BRN G is formally defined as $K(G) = \{K_{v_i}(Q_{x_{v_i}}) \in Z_{v_i}\ \forall v_i \in V\}$.

The discrete evolution of the entity v_i at level x_{v_i} is inferred by the parameter $K_{v_i}(Q_{x_{v_i}})$ using the evolution operator $\Rsh$ following the rule:

$$x_{v_i} \Rsh K_{v_i}(Q_{x_{v_i}}) = \begin{cases} x_{v_i} + 1 & \text{if } x_{v_i} < K_{v_i}(Q_{x_{v_i}}); \\ x_{v_i} & \text{if } x_{v_i} = K_{v_i}(Q_{x_{v_i}}); \\ x_{v_i} - 1 & \text{if } x_{v_i} > K_{v_i}(Q_{x_{v_i}}). \end{cases}$$

Firing Rule

A transition $t \in T$ is enabled and may fire (represented as $m[t\rangle$) if and only if $m(p) \geq f(p,t)\ \forall p \in {}^\circ t$.

The markings of the Petri Net after firing are satisfied as $m'(p) = m(p) - f(p,t) + f(t,p)\ \forall p \in P$.

Cycle

A cycle is an array of markings of length n, formed from the firing of more than one transition, such that $m_0(p) = m_n(p)\ \forall p \in P$.

Deadlock

A deadlock is a marking in the Reachability Graph from which no transition is enabled or live, that is $\neg(m[t\rangle)\ \forall t \in T$ holds.
